# Interleukin-37 Inhibits Colon Carcinogensis During Chronic Colitis

**DOI:** 10.3389/fimmu.2019.02632

**Published:** 2019-11-08

**Authors:** Steffeni Mountford, Andrea Ringleb, Rahel Schwaiger, Doris Mayr, Sebastian Kobold, Charles A. Dinarello, Philip Bufler

**Affiliations:** ^1^Department of Pediatrics, Dr. von Hauner Children's Hospital, Ludwig-Maximilians-University, Munich, Germany; ^2^Institute of Pathology, Ludwig-Maximilians-University, Munich, Germany; ^3^Center for Integrated Protein Science Munich (CIPSM), Division of Clinical Pharmacology, Klinikum der Ludwig-Maximilians-Universität München, Munich, Germany; ^4^German Consortium for Translational Cancer Research (DKTK), Partner Site Munich, Germany; ^5^Department of Medicine and Immunology, University of Colorado School of Medicine, Aurora, CO, United States; ^6^Department of Pediatric Gastroenterology, Nephrology and Metabolic Diseases, Charité Universitätsmedizin Berlin, Berlin, Germany

**Keywords:** chronic colitis, carcinogenesis, cytokines, inflammation, IL-37

## Abstract

Inflammatory bowel disease increases the risk of developing colon cancer. Interleukin (IL-) 37 is a fundamental inhibitor of innate immunity by reducing systemic and local inflammation. IL-37 protein is expressed in healthy and diseased bowel and liver tissue. Here, we tested whether transgenic expression of human IL-37 protects IL-10 deficient (IL-10KO) mice from chronic colitis. IL-37tg mice were crossbred with IL-10KO mice. Homozygous IL-10KO/IL-37tg and IL10KO drank 2% dextran sulfate sodium (DSS) in water for 5 days to induce mild colitis. Colon carcinogenesis was triggered by intragastric administration of celecoxib. Endpoints were clinical parameters of colitis, cytokine responses in LPS-stimulated whole blood and explanted colon specimen and qPCR analysis of colon biopsies. Colon inflammation and number of adenoma—carcinoma were analyzed by histology. During the DSS-induction phase IL-10KO and IL-10KO/IL-37tg mice had a similar weight loss due to mild acute colitis. From day 115 there was a significantly improved weight gain in IL-10KO/IL37-tg mice, though colon length was similar. After *ex vivo* LPS stimulation whole blood of IL-10KO/IL-37tg compared to IL-10KO mice released less IL-6, IL-17, IFNγ, and TNFα and *ex vivo* colon cultures showed reduced IL-6 production both indicative of reduced inflammatory conditions under the influence of IL-37. Six out of 10 IL-10KO mice developed colon adenoma and carcinoma. Only one adenoma but no carcinoma was detected in colons of IL-10KO/IL-37tg mice. In conclusion, IL-37 transgene expression protects IL-10KO mice from colon carcinogenesis. It remains unclear whether IL-37 has direct tumor suppressing properties.

## Introduction

Sustained intestinal inflammation in inflammatory bowel disease (IBD), particularly in ulcerative colitis, is associated with an increased risk for developing colon cancer (1, 2). Even in the absence of clinically overt colitis, inflammatory pathways are involved in the pathogenesis of colorectal cancer (3).

Cytokines play a key role in modulating the innate and intestinal immune response. An imbalance of pro- and anti-inflammatory cytokine release in IBD leads to the disruption of an otherwise tightly controlled immune balance of the gut (4). Cytokines of the IL-1 family exhibit pro- and anti-inflammatory properties (5). The recently discovered IL-1 family member Interleukin (IL-) 37 (formerly known as IL-1H4/IL-1F7b) is a fundamental inhibitor of innate immunity by reducing systemic and local inflammation (6). IL-37 limits the production of cytokines induced by IL-1 and Toll-like receptors (TLR) (7). IL-37 exhibits intra- and extracellular functionality and it has been identified that the IL-37 receptor is composed of IL-18 receptor 1 and single Ig IL-1R-related molecule (SIGIRR) (8–10). Upon caspase-1 cleavage, IL-37, similar to IL-33, translocates to the nucleus (8). Intracellular IL-37 binds Smad3, an important signaling molecule of the TGFß-pathway (7, 11). The inhibition of Smad3 activation reduces the anti-inflammatory properties of IL-37 indicating a functional interaction of both proteins (7).

IL-37 transcripts have been reported in various human tissues such as lymph node, bone marrow, lung, testis and uterus (12). Protein expression has been shown in human blood monocytes, tonsil B cells, plasma cells, breast carcinoma, gut mucosa and liver (7, 12–16). Increased levels of IL-37 mRNA and circulating IL-37 protein have been reported in several human disease conditions (14, 17–21). IL-37 is highly expressed in gut epithelia and submucosal lymphoid cells from patients with inflammatory bowel disease but low in biopsies from healthy subjects (14, 17). Despite epithelial expression, presence of IL-37 in the immune cell compartment is sufficient to protect mice from acute DSS-induced colitis (22).

Recent findings demonstrate that mice overexpressing human IL-37 (IL-37tg) are protected against LPS-induced sepsis (7) and a variety of other models of inflammation including DSS-induced acute colitis (22–25).

IL-10 is a key mediator of gut homeostasis and IL-10 deficient mice (IL10KO) develop spontaneous enterocolitis in response to resident enteric bacteria (23, 26). The histological findings are similar to those of human IBD (23, 27). In analogy, the inborn deficiency of anti-inflammatory IL-10 or its receptor was shown to induce early onset IBD in infants (28). Similar to humans with IBD, IL-10KO mice develop colon cancer in the course of chronic colitis (24).

In the present study, we tested whether transgene IL-37 expression not only protects IL-10KO mice against chronic colitis but also against colon carcinogenesis.

## Materials and Methods

### Chemicals and Reagents

All reagents were purchased from Sigma-Aldrich GmbH (Munich, Germany) unless indicated.

### Animals

Animals were housed in specific-pathogen free condition at a controlled temperature with light/dark cycles with free access to food and water and were acclimatized for 2 weeks before being studied. C57BL/6J mice expressing human IL-37 have been described previously (7). IL-10KO mice were cross-bread with C57BL/6J-IL-37tg mice to create IL-10KO/IL-37tg mice. IL-10KO were obtained from Charles River Inc. (Boston, MA, USA). Homozygous IL-10KO/IL-37tg mice were selected by genotyping. Experiments were conducted on 6–8 week old male and female mice (IL10KO: 4 male, 9 female; IL10KO/IL37tg: 3 male, 6 female). Animal protocols were approved by the review board of the Federal Government of Bavaria, Germany (Az. 55.2.1.54-2532-77-11).

### Induction of Chronic Colitis and Colon Carcinogenesis

IL-10KO mice did not develop significant spontaneous colitis in our animal facility. Therefore, colitis was induced by DSS (2 % DSS wt/vol, 36,00–50,00 kDa; MP Biomedicals, Eschwede, Germany) administered in drinking water *ad libitum* for 5 days at the age of 6 weeks. Cyclooxygenase inhibitor 2 celecoxib (500 μg per mouse per day) was applied by gastric gavage on day 7, 10, and 13 to trigger colon carcinogenesis as described (25, 29) (Supplementary Figure 1). Clinical severity of colitis (stool consistency, weight loss, and fecal blood) were recorded daily for the first 15 days and three times weekly thereafter. Percent weight changes in relation to baseline were scored as follows: 0, no weight loss; 1, 1–5%; 2, 5–10%; 3, 10–15%; 4, >15%. Stool consistency was scored as follows: 0, well-formed pellets; 2, pasty and semi-formed stools; and 4, liquid stools that adhered to the anus. Rectal bleeding was scored as follows: 0, no blood using haemoccult (Beckman Coulter, Palo Alto, CA, USA); 2, positive haemoccult; and 4, gross bleeding. Deceased mice did not fulfill the criteria for premature exclusion from the experiments but died unexpectedly overnight.

### Colon Histology

On day 171 after colitis induction with DSS, mice were bled by intracardial puncture and subsequently sacrificed. After euthanization colon and spleen were dissected, and measured in length and weight.

Whole colons were rolled from distal to proximal end before being fixed in 4% PFA and embedded in paraffin for histological evaluation. Hematoxylin/eosin (H&E) and Ki-67 (Dako, 1:150) staining of 5 μm colon slices were performed according to standard protocols. Colon histology was evaluated by a blinded pathologist according to the scoring system shown in Table 1.

**Table 1:** Histological classification.

**Points**	**Classification**	**Description**
0	I1/T0	Low grade inflammation, no tissue damage
1	I2/T0	Medium grade inflammation, no tissue damage
2	I2/T2	Medium grade inflammation, tissue damage
3	I3/T2	High grade inflammation, tissue damage
4	I3/T3	High grade inflammation, extensive tissue damage
6	RH	Regenerative hyperplasia
8	A	Adenoma
10	PT1	PT1 Carcinoma—invades submucosa
12	PT2	PT2 Carcinoma—Invades muscularis propria

*Histology of colon sections were assessed by a blinded pathologist according to the classification scheme*.

To evaluate histological inflammation of the entire colon whole explanted colon specimen were rolled up from distal to proximal part (“Swiss roles” citation), embedded in paraffin and stained with HE or Ki-67.

Colon specimen were snap frozen in liquid nitrogen for RNA extraction. Histology of colon sections was assessed by a blinded pathologist (D.M.).

### Colon Culture

Specimen from proximal, medium, and distal part of the colon were obtained using a 3 mm dermal punch biopsy device (Stiefel, Munich, Germany) and transferred to a 48-well plate containing 500 μl of cell culture medium (RPMI 1640, FCS 10%, penicillin/streptomycin). After 24 h, supernatants were collected for measurement of spontaneous cytokine release.

### Mouse Whole Blood Culture

Heparinized whole blood was diluted 1:5 in serum free RPMI 1640, added to a 96-well round bottom plate and incubated with or without 1 μg/ml LPS (E. coli 055:B5). After 24 h, supernatants were collected for cytokine measurement.

### Cytokine Measurement

IL-6 was measured by ELISA (BD Biosciences, Heidelberg, Germany). IL-1β, IL-17, IFN-γ, and TNFα were analyzed by BioplexAssay (Biorad, Munich, Germany).

### RNA Isolation and Quantification

Total RNA was isolated from 30 mg of snap frozen colon tissue using RNeasy Mini Kit (Qiagen, Hilden). RNA samples (1 μg) were reverse transcribed using SuperScript™ II Reverse Transcriptase (Invitrogen, Carlsbad, CA, USA). Gene expression levels were measured by quantitative PCR (SYBR Green Supermix, Biorad). Gene specific primers were designed using PrimerExpress and ordered from Eurofins MWG (Ebersberg, Germany) with purification grade HPLC. qRT-PCR reactions were performed in triplets in a 96-well format (BioRad iCycler). Fold changes of mRNA expression were calculated and normalized to TATA-box binding protein gene expression using the ΔΔCt-method. The sequences of the gene specific primers used are listed in Table 2.

**Table 2:** Gene-specific primer sequences.

**Gene**	**Forward primer**	**Reverse primer**
*Foxp3*	5′-ATC CCC CTC TAG CCA GTC CAC-3′	5′-AGT TGC CGG GAG AGC TGA AT-3′
*Rorγt*	5′-TAC CCT ACT GAG GAG GAC AGG-3′	5′-TTG ACA GCA TCT CGG GAC A-3′
*Il-22*	5′-TGC GAT CTC TGA TGG CTG-3′	5′-CCT TAG CAC TGA CTC CTC GG-3′
*Il-22bp*	5′-CAG CGG ATC ACC CAG AAG TT-3′	5′-GCG GTT TGA TGG TAG TGT GC-3′
*Il-22r*	5′-GCA CCT CTG ACA CCG TCT AC-3′	5′-GGT TTG ATG GTA GTG TGC TGC-3′
*Stat3*	5′-TGT GAC ACC ATT CAT TGA TGC AG-3′	5′-ACA CTC CGA GGT CAG ATC CA-3′
*IL-17a*	5′-CTC AAA GCT CAG CGT GTC CA-3′	5′-TTT GCG CCA AGG GAG TTA AA-3′
*Ahr*	5′-GAG TTC TTG TTA CAG GCG CTG A-3′	5′-AGG AAG CTG GTC TGG GGT AT-3′
*Tbet*	5′-CAT GCC AGG GAA CCG CTT AT-3′	5′-ATT GTT GGA AGC CCC CTT GT-3′
*Tbbp*	5′-GCC CGA AAC GCC GAA TAT-3′	5′-CCG TGG TTC GTG GCT CTC T-3′

### Statistical Analysis

Results are expressed as mean ± SEM. All samples were tested for normal distribution by Kolmogorov-Smirnov test and analyzed by unpaired *t*-test. Samples without normal distribution were analyzed by Mann-Whitney test. Statistical analysis was performed with Prism 5 Version 5.0d for Macintosh.

## Results

### Transgene IL-37 Expression Is Associated With Improved Clinical Outcome of Chronic Colitis in IL-10KO Mice

**Figure 1:**
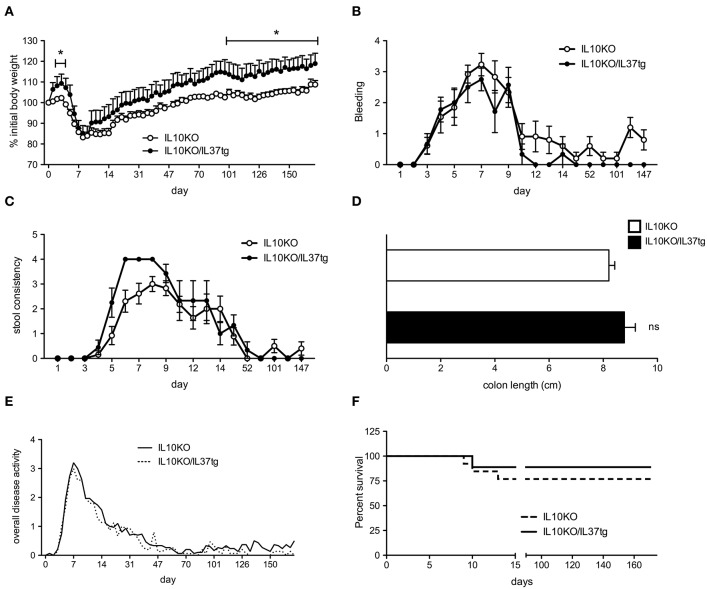
Transgene IL-37 expression is associated with improved clinical outcome of chronic colitis in IL-10KO mice. Mild colitis was induced in IL-10KO/IL-37tg and IL-10KO mice which were scored in regards to **(A)** percent weight change (0: none, 1: −5%, 2: 5–10%, 3: 10–15%, 4: >15%), **(B)** blood in the stool (0: normal, 2: slight bleeding, 4: gross bleeding), **(C)** stool consistency (0: normal, 2: loose stool, 4: watery diarrhea) and **(D)** colon length **(E)** Overall disease activity: Clinical severity of colitis (stool consistency, weight loss and fecal blood) were recorded daily for the first 15 days and three times weekly thereafter **(F)** Overall survival. Open circles/bars: IL10KO (*n* = 10), closed circles/bars: IL-10KO/IL-37tg (*n* = 5). ^*^p < 0.01. Statistical significance tested with Mann-Whitney-Test.

Previous studies revealed that mice expressing human IL-37 are protected from acute DSS colitis (22). We therefore hypothesized that IL-37 plays a protective role in the IL-10KO chronic colitis model and subsequent colon carcinogenesis. IL-37tg mice were cross bred with IL-10 deficient mice to generate IL-10KO/IL-37tg mice. Since IL-10KO mice did not develop spontaneous colitis in our animal facility, we induced mild colitis by administering low dose DSS for 5 days, followed by three administrations of celecoxib via gastric gavage as described (29). During initial DSS treatment IL-10KO/IL-37tg mice showed an increased body weight compared to IL-10KO mice (Figure 1A). After 5 days, both IL-10KO and IL-10KO/IL-37tg mice significantly lost body weight. From d100 IL-10KO/IL-37tg mice showed a better weight gain compared to IL-10KO mice (Figure 1A). Initial gastrointestinal bleeding induced by DSS was similar in both groups (Figure 1B). From day 12, IL-10KO/IL-37tg mice showed an overall trend toward less bleeding. Stool consistency score was only mildly elevated and similar in both groups indicating low-grade colon inflammation as anticipated and planned for the study (Figure 1C). Stool consistency was normal in both groups from day 50. At the end of the experiment, the length of explanted colons was similar between the groups (Figure 1D). Overall disease activity was similar in both groups (Figure 1E). Three mice in the IL-10KO group died or required sacrifice after the induction of colitis by DSS. One mouse of the IL-10KO/IL-37tg group died (Figure 1F).

### Effect of Transgenic IL-37 on Blood Differentiation and Cytokine Release in Whole Blood Cultures of IL-10KO Mice During Chronic Colitis

**Figure 2:**
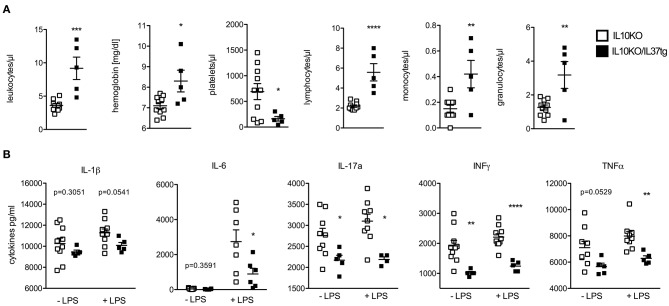
Effect of transgene IL-37 on blood differentiation and cytokine release in whole blood cultures of IL-10KO mice during chronic colitis. **(A)** Heparinized blood samples were analyzed by automatic blood smear differentiation at the time of colon removal. **(B)** Heparinized mouse whole blood samples were stimulated with LPS (1 μg/ml) or not. Cytokines were measured in supernatants after 24 h by multiplex array or Elisa (IL-6). Data are expressed as the mean (SEM). Open squares: IL-10KO mice (*n* = 10), closed squares: IL-10KO/IL-37tg mice (*n* = 5). ^*^p < 0.05, ^**^p < 0.01, ^***^p < 0.001, ^****^p < 0.0001. Statistical significance tested with Mann-Whitney-Test.

At the time of colon removal blood samples were analyzed by an automatic cell counter. As compared to IL-10KO mice leukocytes, hemoglobin, lymphocytes, and granulocytes were increased in the IL-10KO/IL-37tg mice, whereas the platelet count was lower (Figure 2A). LPS-induced pro-inflammatory cytokines IL-17a, IL-6, IFNγ, and TNFα in *ex vivo*, whole blood stimulation assays were decreased in IL-10KO/IL-37tg mice. Spontaneous IL-17a and IFNγ secretion was also lower in whole blood assays in IL-10KO/IL-37tg mice. IL-1β secretion showed a trend in reduction in spontaneous and stimulated whole blood assay of IL10KO/IL37tg mice (Figure 2B).

### Spontaneous Cytokine Release From Colon Biopsies

**Figure 3:**
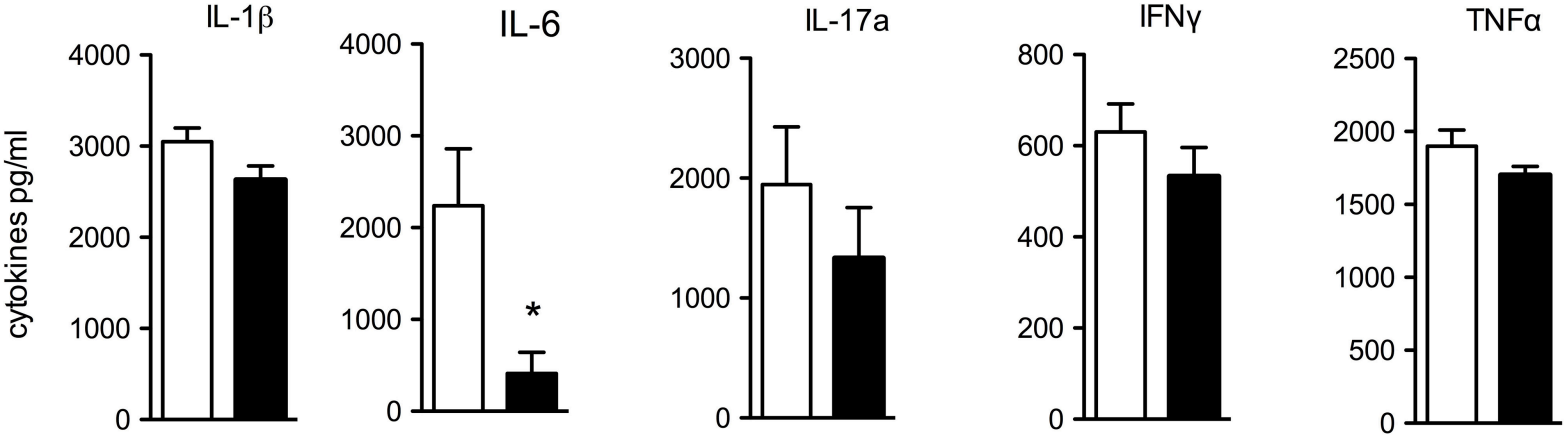
Spontaneous cytokine release from colon biopsies. Transmural biopsies from macroscopically non-inflamed areas of the proximal colon were taken at the time of colon removal during chronic colitis and incubated in RPMI for 24 h. Supernatants were tested for spontaneous cytokine release. Cytokines were measured by multiplex array and Elisa (IL-6). Data are expressed as the mean (SEM). Open bars: IL-10KO mice (*n* = 10), closed bars: IL-10KO/IL-37tg mice (*n* = 5). ^*^p < 0.05. Statistical significance tested with Mann-Whitney-Test.

Next, we investigated the spontaneous cytokine release from macroscopically non-inflamed proximal colon specimen by incubating punch biopsies for 24 h in RPMI and testing for spontaneous cytokine release. IL-6 was significantly reduced in supernatants of IL-10KO/IL-37tg colon biopsies compared to IL-10KO mice (Figure 3). Concentrations of pro-inflammatory IL-1β, IL-17a, IFNγ, and TNFα trended to be lower.

### Reduced Inflammation and Colon Carcinogenesis in IL-10KO/IL-37tg Mice in the Course of Chronic Colitis

**Figure 4:**
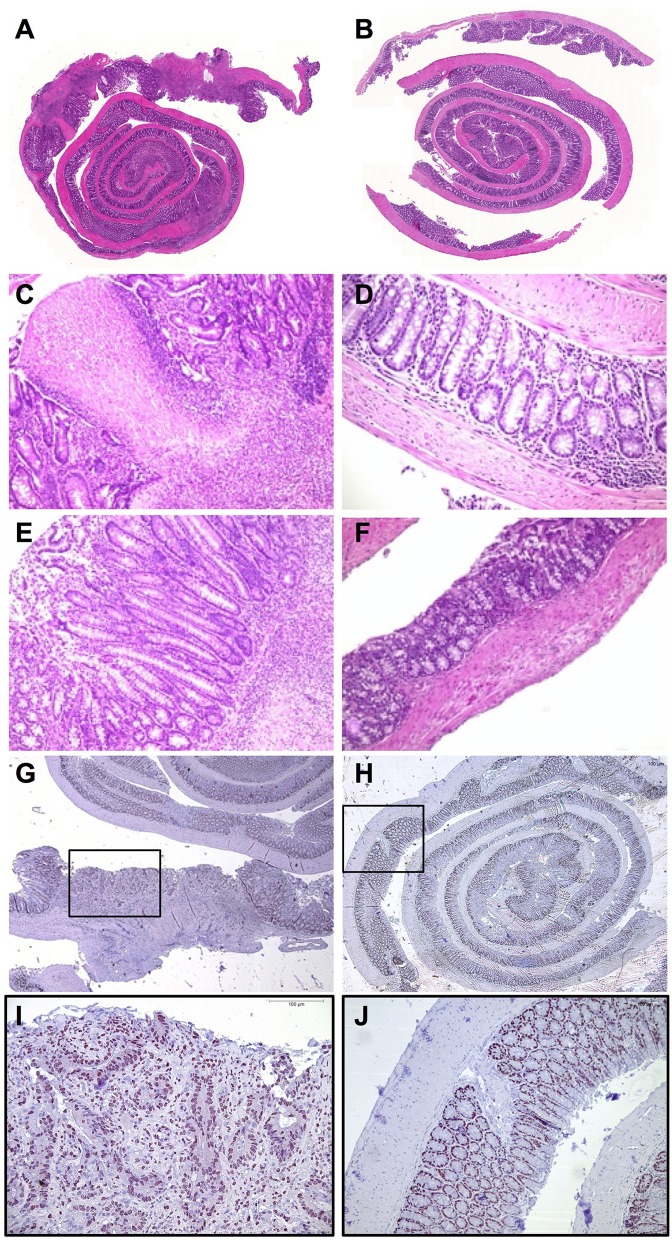
Reduced inflammation and colon carcinogenesis in IL-10KO/IL-37tg mice in the course of chronic colitis. Whole explanted colons from IL-10KO mice **(A)** and IL-10KO/IL-37tg mice **(B)** were rolled up from distal to proximal (“swiss role”), transferred to formaldehyde solution for 24 h and subsequently embedded in paraffin. Cross-sections of full-length bowel specimen underwent HE-staining **(A–F)**. **(C)** Ulceration and high-grade inflammation of IL-10KO mouse. **(D)** Low-grade infiltration IL-10KO/IL-37tg. **(E)** Regenerative Hyperplasia with high-grade inflammation in IL-10KO mice. **(F)** Mild- infiltration in IL-10KO/IL-37tg mouse. Cross-sections of full-length bowel specimen underwent Ki-67-staining **(G,H)**. PT2 carcinoma in IL-10KO mouse **(G,I)**. Healthy tissue of IL-10KO/IL-37tg mouse **(H,J)**.

**Figure 5:**
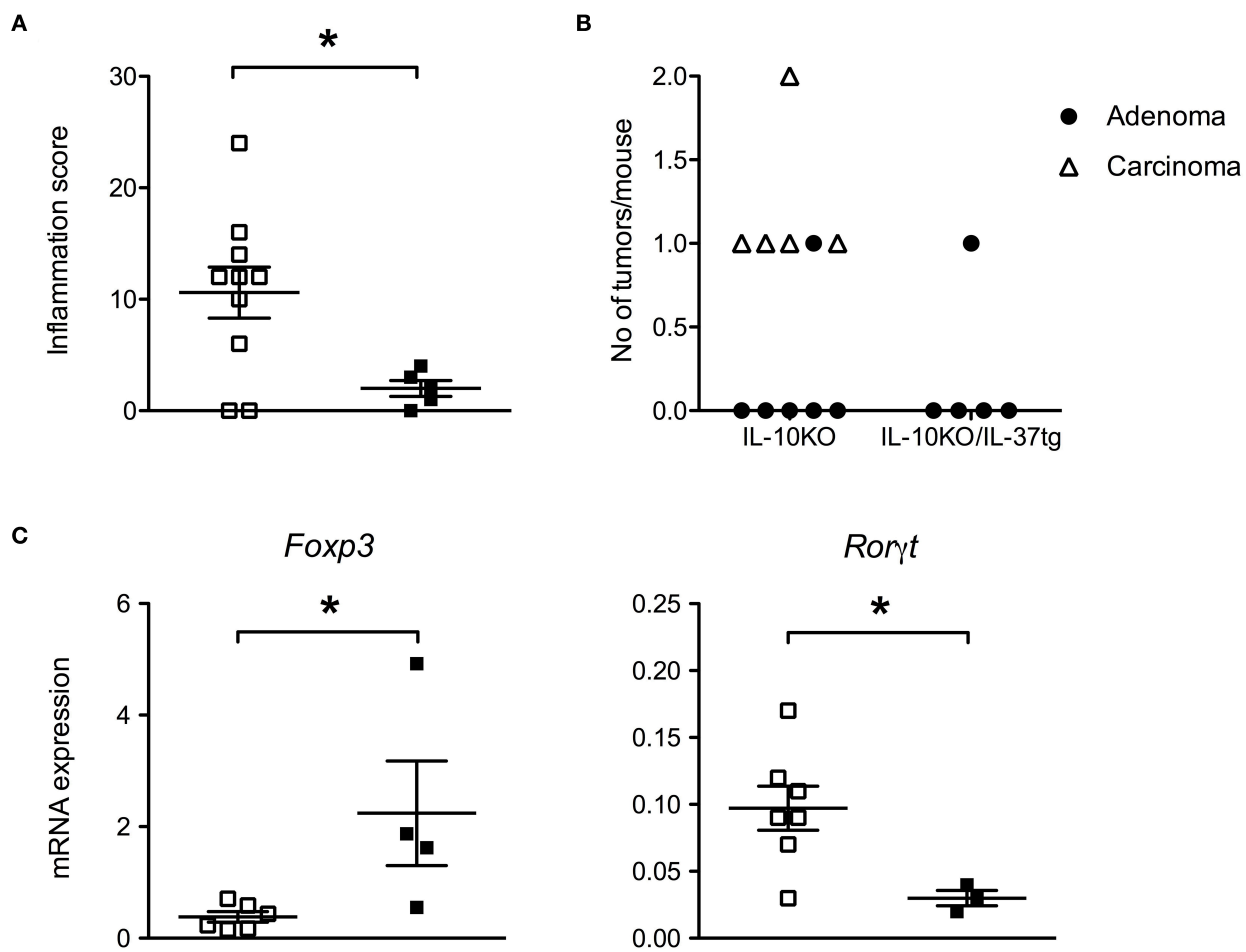
Transgenic IL-37 inhibits colon carcinogenesis. **(A)** Inflammation score, **(B)** total number of adenomas and carcinomas (IL-10KO *n* = 10, IL 10KO/IL-37tg *n* = 5) and **(C)** gene expression in macroscopically normal areas of distal colons. The grade of inflammation and number of tumors were assessed by histology. Gene expression was normalized according to TBP expression. Open squares: IL-10KO (*n* = 7), closed squares: IL-10KO/IL-37tg (*n* = 4). ^*^p < 0.05. Statistical significance tested with Mann-Whitney-Test.

The entire colon of IL-10KO mice shows marked changes in mucosal architecture, especially in the proximal colon compared to the specimen of IL-10KO/IL-37tg mice (Figures 4A,B). IL-10KO mice show higher-grade inflammation, necrosis with concomitant erosions, ulcerations and crypt degeneration (Figures 4C, 5A) as well as regenerative hyperplasia (Figure 4E). In some mice, there was increased tissue fibrosis and inflammation reaching the submucosal structures (Supplementary Figure 2A).

In contrast, IL-10KO/IL-37tg mice only show low-grade mucosal inflammation with mild lymphoid infiltration (Figures 4D,F, 5A). The overall tissue structure is intact with only few minor local erosions (Supplementary Figure 2B).

Ki-67 protein is a cellular marker for proliferation (30) and its staining pattern corresponds in IL-10KO colons to the severely impaired tissue architecture (Figure 4G). Increased numbers of Ki-67-positive cells extend into the muscularis mucosa layer and are associated with adenomas and carcinomas in the proximal colon (Figure 4I). The tissue structure is intact in IL-10KO/IL-37tg (Figure 4H) and regular numbers of Ki-67 positive cells are only detected in crypts (Figure 4J).

### Transgenic IL-37 Inhibits Colon Carcinogenesis

Six out of 10 IL-10KO mice developed adenomas or carcinomas. One IL-10KO mouse developed two carcinomas (Figure 5B). No carcinoma and only one adenoma were detected in IL-10KO/IL-37tg mice (Figure 5B). All adenoma and carcinoma in IL-10KO mice are localized in the proximal part of the colon.

In order to determine whether human IL-37 expression in IL-10KO mice is associated with a tumor protective immune status in the gut, we analyzed the levels of selected mRNA's linked to tumor formation. In transmural biopsies from macroscopically normal areas of the distal colon, gene expression of Rorγt was decreased and Foxp3 was increased in IL-10KO/IL-37tg mice compared to IL-10KO mice (Figure 5C). Other inflammation associated genes as IL-22, IL-22BP, IL-22R, IL-17a, Ahr, T-bet, and Stat3 showed no difference in expression level (Supplementary Figure 3).

## Discussion

Inflammatory bowel disease is associated with an increased risk of developing colorectal cancer. Here we investigated whether transgene expression of human IL-37 protects against colon inflammation and carcinogenesis in IL-10KO mice. Crossbred IL-10KO/IL-37tg mice showed less weight loss from day 100 during chronic colitis compared to IL-10KO mice. LPS-induced cytokine release in cultured whole blood as well as spontaneous cytokine release from *ex vivo* cultures was reduced in IL-37-expressing mice. There was a lower severity of histologic colon inflammation in IL-10KO/IL-37tg mice and colon carcinogenesis was only detected in IL-10KO mice.

The development of colitis in IL-10KO mice depends on the local microbial environment (26). In our animal facility IL-10KO mice did not develop spontaneous colitis. In order to trigger the development of chronic colitis we induced mild colitis by administering a low dose of DSS followed by intragastric application of celecoxib as described by others (29). The increased weight gain during the induction phase of colitis, the trend toward reduced bleeding from day 12 and the better weight gain from day 100 in IL-10KO/IL-37tg mice is consistent with our previous observation showing that transgene overexpression of IL-37 protects mice from acute DSS-induced colitis (22). Despite mild inflammatory activity of colitis some mice died during the chronic phase and there was a higher mortality in IL-10KO mice.

Importantly, we show that transgene IL-37 is active in IL-10KO mice. An antibody to the IL-10 receptor did also not counteract the effect of IL-37 in the model of acute DSS-induced colitis (22). This clearly indicates that the immune-regulatory function of IL-37 is not mediated by IL-10.

The lower clinical severity of chronic colitis in IL-10KO/IL-37tg mice correlated with elevated hemoglobin indicating less intestinal bleeding; we also observed higher counts for lymphocytes, granulocytes and leucocytes. In contrast, platelet counts are significantly higher in IL-10KO mice reflecting a higher level of systemic inflammation as seen in children during chronic colitis (31).

The initial hallmark of IL-37 activity was the reduced secretion of pro-inflammatory cytokines in transfected cells and the protection of IL-37tg mice from LPS-induced shock (7, 10). We recently showed that PBMC isolated from the peripheral blood of children with IBD exhibit an increased responsiveness to LPS indicating a higher level of innate immune activity (32). At the time of *ex vivo* colon cultures, we therefore also cultured whole blood from IL-10KO and IL-10KO/IL-37tg mice in the presence of LPS. Indeed, we found that IL-37 overexpression is not only associated with reduced baseline but also with reduced LPS-induced secretion of prototypic inflammatory markers such as IL-6, IL-17a, IFNγ, and TNFα from whole blood cultures.

Since chronic inflammation in IBD triggers colon carcinogenesis in humans (1, 33), we next measured the *ex vivo* spontaneous pro-inflammatory cytokine release from punch biopsies of the colon. Since already subtle inflammation is associated with an increased risk for colon cancer (3), we investigated the biopsies from macroscopically non-inflamed areas of the proximal colon and found a significantly reduced IL-6 secretion in colon specimen isolated from IL-10KO/IL-37tg mice compared to IL-10KO mice. Other pro-inflammatory cytokines as IL-1β, IL-17a, IFNγ, and TNFα were also lower but did not reach statistical significance, indicating that IL-37 contributes to an anti-inflammatory microenvironment within the colon.

The subsequent histological evaluation of the entire colon supports these observations. In contrast to bona fide IL-10KO mice, IL-10KO mice expressing IL-37tg show only low-grade mucosal inflammation. Unexpectedly, we detected no carcinoma and only one adenoma in IL-10KO/IL-37tg mice, whereas 6 out of 10 IL-10KO mice developed adenoma or carcinoma.

Ongoing stimulation of epithelial proliferation in an inflammatory environment is key for the pathogenesis of colorectal cancer (33, 34). Indeed, we found markedly increased numbers of Ki-67-positive epithelial cells in colons of IL-10KO mice, which extended into the muscularis mucosa layer and were associated with adenoma and carcinoma of the proximal colon. In biopsies of the distal colon without adenomas and carcinomas, there was a lower level of gene expression of Rorγt and higher gene expression of Foxp3 in IL-10KO/IL-37tg mice indicating a tumor protective immune status associated with transgene IL-37 expression. In addition, the right-sided location of colon carcinoma in IL-10KO mice indicates that transgene IL-37 expression additionally impacts bile acid metabolism which has been shown to be involved with PSC-associated colon carcinogenesis (35).

Consistent with our observation Gao et al. reported profound anti-tumor activity of IL-37 after adenoviral expression in established mouse fibrosarcoma tumors, which was abrogated in nude and SCID mice (36). More recent reports indicate that IL-37 induces autophagy in hepatocellular carcinoma by inhibiting the PI3K/AKT/mTOR pathway and suppresses tumor activity in renal cell and small lung cell carcinoma (37, 38). Furthermore, Yan et al. showed a reduced tumor formation in mice overexpressing IL-37 after treatment with azoxymethane and DSS by suppressing the β-catenin pathway. In human colorectal cancer reduced IL-37 expression was associated with disease progression and poor outcome (39).

In summary, we show that transgene expression of human IL-37 protects IL-10KO mice from colon carcinogenesis. Further studies are needed to clarify whether IL-37 has direct tumor suppressing properties or rather acts indirectly by modulating inflammation. Additional factors might also be associated with IL-37 transgene expression, i.e., changes of the intestinal microbiota and subsequent changes of bile acid metabolism, as discussed for human PSC and colorectal cancer (40, 41). Future studies are needed to unravel the potential of IL37 upregulation in the gut as a possible therapeutic approach to reduce inflammation and colon carcinogenesis.

## Data Availability Statement

All datasets generated for this study are included in the article/Supplementary Material.

## Ethics Statement

The animal study was reviewed and approved by review board of the Federal Government of Bavaria, Germany (Az. 55.2.1.54-2532-77-11).

## Author Contributions

SM, AR, RS, and PB performed and evaluated the experiments. DM conducted histological evaluation. SK and CD were involved in planning of the study, contributed to the evaluation and discussion of the results, and revised the manuscript. SM and PB wrote the manuscript.

### Conflict of Interest

The authors declare that the research was conducted in the absence of any commercial or financial relationships that could be construed as a potential conflict of interest.

## Abbreviations

IBD: inflammatory bowel disease
IL-10KO: IL10 deficient mouse
IL-: Interleukin
LPS: Lipopolysaccharide
PBMC: peripheral blood mononuclear cells
TLR: Toll like receptor.
