# Supplementary material for: Interleukin-37 Inhibits Colon Carcinogensis During Chronic Colitis
Source: Front Immunol. 2019 Nov 8;10:2632. doi: 10.3389/fimmu.2019.02632 (PMC6857648; doi:10.3389/fimmu.2019.02632)
Supplement: Supplementary file 1 [file Data_Sheet_1.PDF]

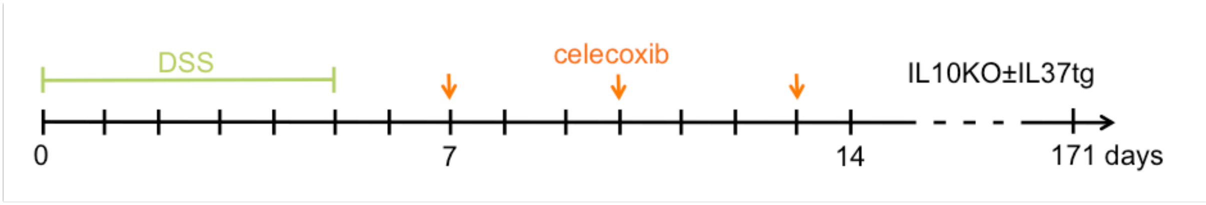

Supplementary Figure 1: Induction of colitis and tumorigenesis by DSS and celecoxib.

Mild colitis was induced by DSS (2% DSS wt/vol, 36,00 – 50,00 kDa; MP Biomedicals) administered in drinking water ad libitum for five days at the age of 6 weeks. Cyclooxygenase inhibitor 2 celecoxib (500 µg/mouse/application) was applied by gastric gavage on day 7, 10 and 13 to trigger colon carcinogenesis

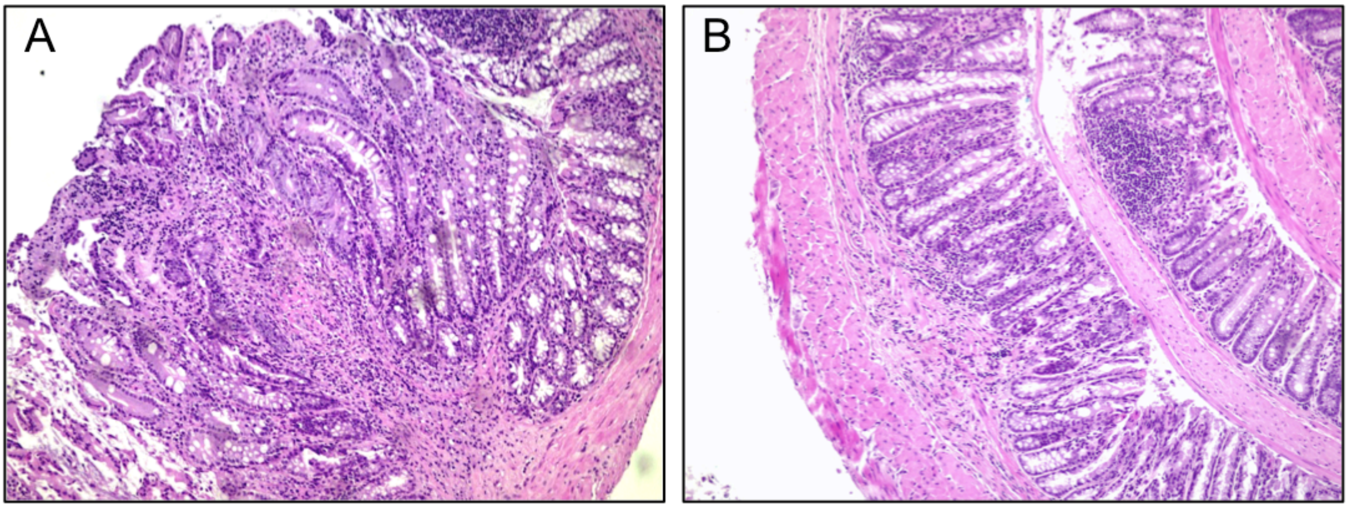

Supplementary Figure 2: Reduced inflammation and intact tissue structures in IL-10KO/IL-37tg mice.

Whole explanted colons from IL-10KO mice (A) and IL-10KO/IL-37tg mice (B) were rolled up from distal to proximal ('swiss role'), transferred to formaldehyde solution for 24 hrs and subsequently embedded in paraffine. Cross-sections of full-length bowel specimen underwent HE-staining. (A) Tissue fibrosis and inflammation reaching the submucosal structures in IL-10KO mouse. (B) Overall intact structures with minor erosions in IL-10KO/IL-37tg mouse.

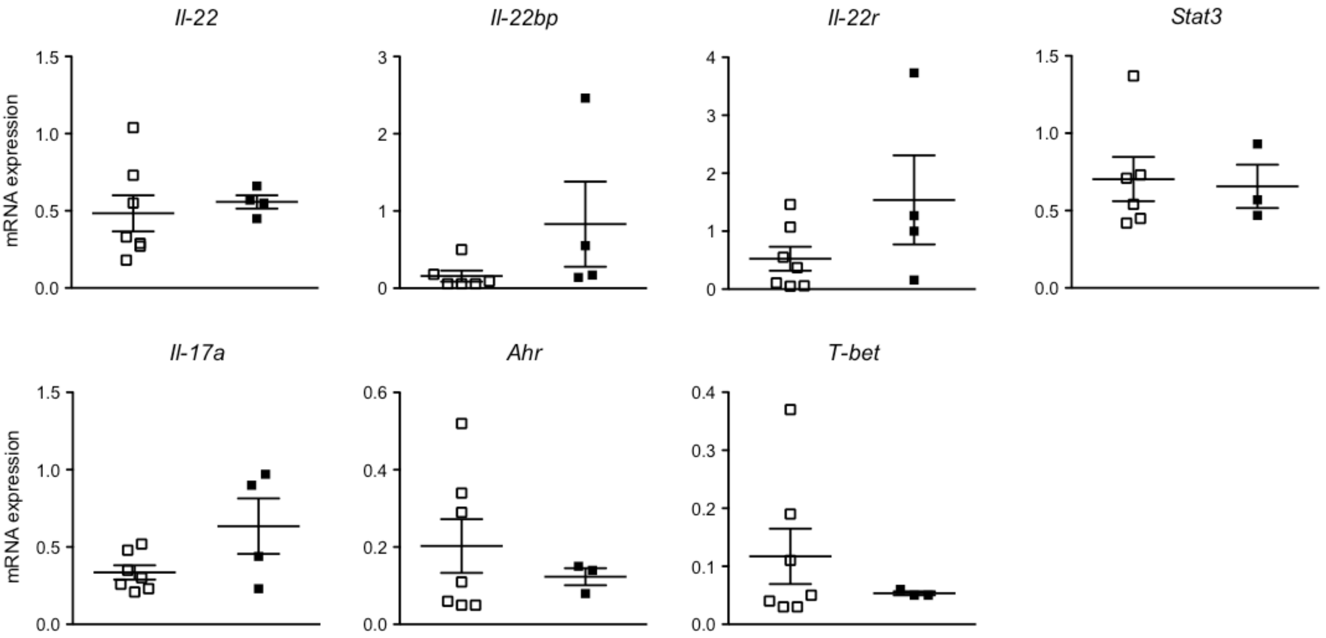

Supplementary Figure 3: Inflammation associated gene expression in distal colon.

Total RNA was extracted from frozen colon tissue and transcribed to cDNA. Gene expression was normalized according to TBP expression. Open squares: IL-10KO (n = 7), closed squares IL-10KO/ IL-37tg (n = 4).
